# A concise drug alerting rule set for Chinese hospitals and its application in computerized physician order entry (CPOE)

**DOI:** 10.1186/s40064-016-3701-4

**Published:** 2016-12-01

**Authors:** Yinsheng Zhang, Xin Long, Weihong Chen, Haomin Li, Huilong Duan, Qian Shang

**Affiliations:** 1 School of Computer Science and Information Engineering, Zhejiang Gongshang University, Hangzhou, 310018 Zhejiang People’s Republic of China; 2 College of Biomedical Engineering and Instrument Science, Zhejiang University, Hangzhou, 310027 Zhejiang People’s Republic of China; 3 Department of Pharmacy, DaYi Hospital, Taiyuan, 030012 ShanXi People’s Republic of China; 4 Children’s Hospital, Institute of Translational Medicine, School of Medicine, Zhejiang University, Hangzhou, 310058 Zhejiang People’s Republic of China; 5 Management School, Hangzhou Dianzi University, Hangzhou, 310058 Zhejiang People’s Republic of China

**Keywords:** Medication-related clinical decision support, Chinese patent medicine, Drug alerting rule, Alert fatigue

## Abstract

**Background:**

A minimized and concise drug alerting rule set can be effective in reducing alert fatigue.

**Objectives:**

This study aims to develop and evaluate a concise drug alerting rule set for Chinese hospitals. The rule set covers not only western medicine, but also Chinese patent medicine that is widely used in Chinese hospitals.

**Setting:**

A 2600-bed general hospital in China.

**Methods:**

In order to implement the drug rule set in clinical information settings, an information model for drug rules was designed and a rule authoring tool was developed accordingly. With this authoring tool, clinical pharmacists built a computerized rule set that contains 150 most widely used and error-prone drugs. Based on this rule set, a medication-related clinical decision support application was built in CPOE. Drug alert data between 2013/12/25 and 2015/07/01 were used to evaluate the effect of the rule set.

**Main outcome measure:**

Number of alerts, number of corrected/overridden alerts, accept/override rate.

**Results:**

Totally 18,666 alerts were fired and 2803 alerts were overridden. Overall override rate is 15.0% (2803/18666) and accept rate is 85.0%.

**Conclusions:**

The rule set has been well received by physicians and can be used as a preliminary medical order screening tool to reduce pharmacists’ workload. For Chinese hospitals, this rule set can serve as a starter kit for building their own pharmaceutical systems or as a reference to tier commercial rule set.

## Background

Computerized physician order entry (CPOE) with medication-related clinical decision support (CDS) is an effective solution to reduce drug-related problems and pharmacist workload (Hammar et al. 2015; Claus et al. 2015). Most medication-related decision support functions, such as dosage checking and drug–drug interaction (DDI) checking, are typically implemented by a set of computerized drug alerting rules. One major problem faced by drug alerting rules is the alert fatigue (Nanji et al. 2014), which is usually caused by highly exhaustive and sensitive rules. Recent related work shows override rates can be as high as 53.6% (Nanji et al. 2014), 87.6% (Topaz et al. 2015), and 93% (Bryant et al. 2013) respectively. To address this issue, lots of work has been focused on constructing minimized and concise drug rule sets. For example, Shah et al. (2006) built a tiered medication knowledge subset from a commercial knowledge base. The subset contains clinical significant drug contraindications, and only interrupts physicians for severe alerts. Phansalkar et al. (2012) developed a minimum set of 15 high-severity, clinically significant DDIs from several commercial knowledge bases. Classen et al. (2011) identified 7 most common DDIs by reviewing multiple sources. The public DDI knowledge base SFINX (Swedish, Finnish, INteraction X-referencing) tiers DDIs according to clinical significance (A-D), which enables threshold settings for automated warnings (Andersson et al. 2015).

## Aim of the study

The aim of this study to build and evaluate a concise rule set suitable for Chinese hospitals. Compared to existing related work, this rule set not only covers the western medicine, but also includes various Chinese patent medicine (CPM) that is extensively used by Chinese hospitals. For example, a typical Chinese hospital (DaYi Hospital, ShanXi Province, China) uses 1981 drugs, and 462 (23.3%) are Chinese patent medicine.

### Ethical approval

 This study was approved by the medical ethics committee of DaYi Hospital. All collected data have been de-identified by the information department of the hospital.

## Methods

### Settings and materials

DaYi Hospital was established in 2011 and is the largest general hospital (2600-bed) in ShanXi Province, China. Until 2013, all the drug checking work in DaYi was performed manually by clinical pharmacists. At the drug dispensing time, the pharmacists would inspect medication orders submitted by the physicians. Unqualified orders would be returned to physicians and recorded by the pharmacists. The recorded medication errors between 2011 and 2013 were used to analyze the most frequent and error-prone drug rules. These records are the initial resource for building the concise rule set.

In 2013, we initiated the KTP (Knowledge Translation Platform) project (Zhang et al. 2015). One of KTP’s goals is to build a medication-related CDS for CPOE, in order to help pharmacists reduce work load and assist the drug checking process. At the beginning of KTP, a preliminary question is: whether to develop own medication-related CDS or use a commercial one. Although there are already mature commercial products on the Chinese market, e.g. Wolters Kluwer/Medicom PASS (Prescription Automatic Screening System), we have our own considerations for not choosing such off-the-shelf systems. (1) Although the rule base of commercial products may be much more comprehensive and detailed, it is still necessary to tier and routinely tailor the complete rule set to suit local hospital situations. For pharmacists, there is not much workload advantage over maintaining a local-developed rule set. (2) From the perspective of the KTP project, the pharmaceutical knowledge is an inseparable part of the entire knowledge base. Inside the KTP knowledge base, there are semantic relations between drug and other medical entities. For example, many clinical rules (e.g. *if [Use of Aspirin]* == *true || [Use of Clopidogrel]* == *true, recommend [INR monitor]*) and clinical treatment protocols (predefined order sets or clinical pathways) involves drug entities. If using third-party products, even if the vendors open their knowledge base or provide external access interfaces, the integration and interaction between different systems (e.g. mapping of drug entities across systems) can be complex and effort-taking. Therefore, we decided to develop an own system.

### Information model

**Fig. 1:**
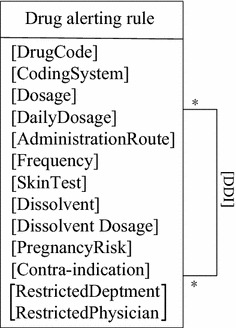
The Information model for drug alerting rules

To implement a computerized rule set, an information model of drug alerting rules is designed (Fig. 1). It defines 11 rule types (Table 1), including dosage (single intake), daily dosage (accumulated intake), administration route, frequency, skin test, dissolvent, dissolvent dosage, DDI, contra-indication, and prescription restriction.

**Table 1:** Drug alerting rule types

Rule type	Description	Example
Dosage	Defines maximum dosage for one medical order	Maximum dosage of Ambroxol injection is 2 doses [Dosage] ≤ 2 doses
Daily dosage	Defines maximum daily accumulated dosage	Maximum daily dosage of ShuXueNing injection (Ginkgo biloba extract) is 4 doses [DailyDosage] ≤ 4 doses
Administration route	Defines allowed administration route	Cobamamide injection should be administrated by intramuscular injection [AdministrationRoute] = {intramuscular}
Frequency	Defines allowed frequency	Ceftriaxone injection frequency should be qd. (1/day) [Frequency] = {qd}
skin test	Defines whether skin test flag should be specified in the medication order, so as to remind the nurses	Lidocaine hydrochloride injection needs skin test [SkinTest] = true
Dissolvent	Defines allowed dissolvent	Dissolvent for pHGF injection can only be 10% glucose injection [Dissolvent] = {10% glucose}
Dissolvent dosage	Defines maximum dissolvent dosage	Dissolvent dosage for iron sucrose injection is 100 ml 100 ml ≤ [DissolventDosage] ≤ 100 ml
Pregnancy risk	Assigns each drug to FDA pregnancy category, which contains five categories: ABCDX. Category X should never be applied to pregnant patients	FDA pregnancy category of Ribavirin is X [PregancyRiskLevel] = X
Drug-drug interaction (DDI)	Defines synergistic, antagonistic, etc. interactions between drugs	Warfarin and Vitamin K have antagonistic interaction Interaction (Warfarin, Vitamin K)
Contra-indication	Defines drug-disease and drug-symptom conflicts	Clopidogrel cannot be used against patients with active peptic ulcer [Contra-indication] = ”[active peptic ulcer] == false && [gastrointestinal hemorrhage] == false”, check passed if result is true
prescription restriction	Restricts the prescription of certain drugs for some departments or physicians	For third-line antibiotics such as Vancomycin, only chief physicians have prescription rights. Pediatrics departments cannot prescribe Vancomycin [RestrictedDeptment] = {pediatrics}, [RestrictedPhyscian] = {ID1, ID2,…}

These rule types are designed according to pharmacists’ drug checking requirements. However, there are also other rule types, such as personalized dosing algorithms (e.g. children or elder patients with different body weights and body surface areas, or patients with renal insufficiency based on creatinine clearance). In the current development phase, we haven’t supported such rules because they require lots of patient context data, such as body weight, body surface area, Crcl rate, etc. These data mostly reside in heterogeneous formats in external systems, such as HIS (Hospital Information System), LIS (Laboratory Information System), EMR (Electronic Medical Record), etc. How to extract high-quality and well-structured data in expected formats from various sources is a non-trivial task. In the next development phase, we will try to solve this data acquisition problem and support more rule types.

### Authoring tool

**Fig. 2:**
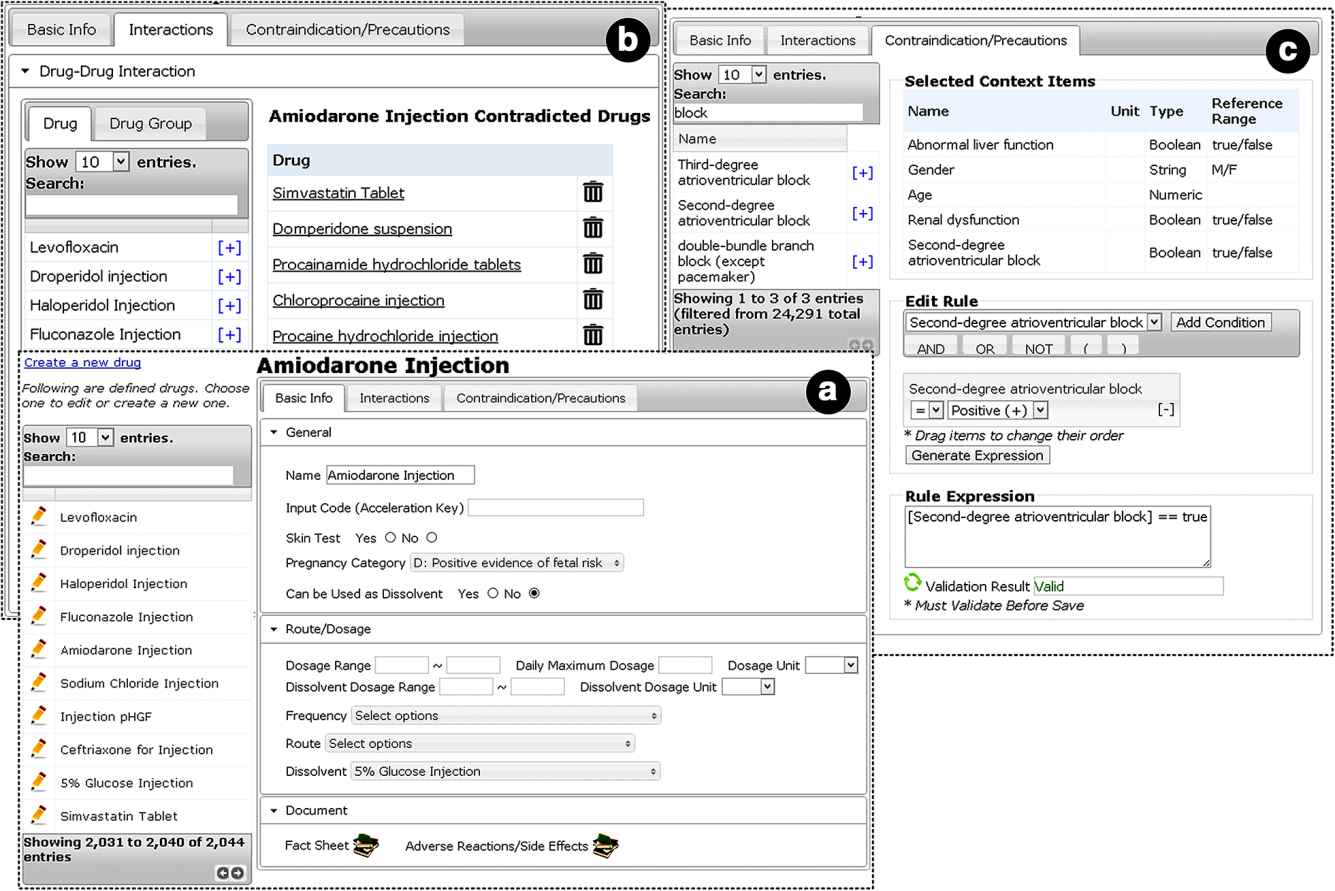
Drug alerting rule authoring tool. **a** Main page for editing drug rules. The *left panel* is the drug list, where user can click one to edit. On the *right side* is the edit area, which contains three tab pages: basic info, interactions and contraindications. Basic info tab page defines basic rules such as skin test, dosage, etc. **b** Tab page for editing drug–drug interactions. Users can select drugs that have interactions with the current one. **c** Tab page for editing contraindication rules. *Left panel* is the context item (e.g. lab test, symptoms, vital signs, etc.) list used to define contraindicated conditions. The *right side* is a table of user-selected context items, and a graphical rule composer, as well as a textual rule expression editor

Based on the above information model, the database schema for drug alerting rules can be decided, and a corresponding rule authoring tool has been developed (Fig. 2). The tool was developed as a web-based application.

## Results

### Drug alerting rule set

Based on the recorded medication errors between 2011 and 2013, the pharmacists used the rule authoring tool to define a rule set that was able to cover the most widely used and error-prone drugs. The first version of the rule set was created in June 2013, and contained 150 drugs. The detailed rule set is provided in “Appendix”.

### Medication-related CDS based on the rule set

**Fig. 3:**
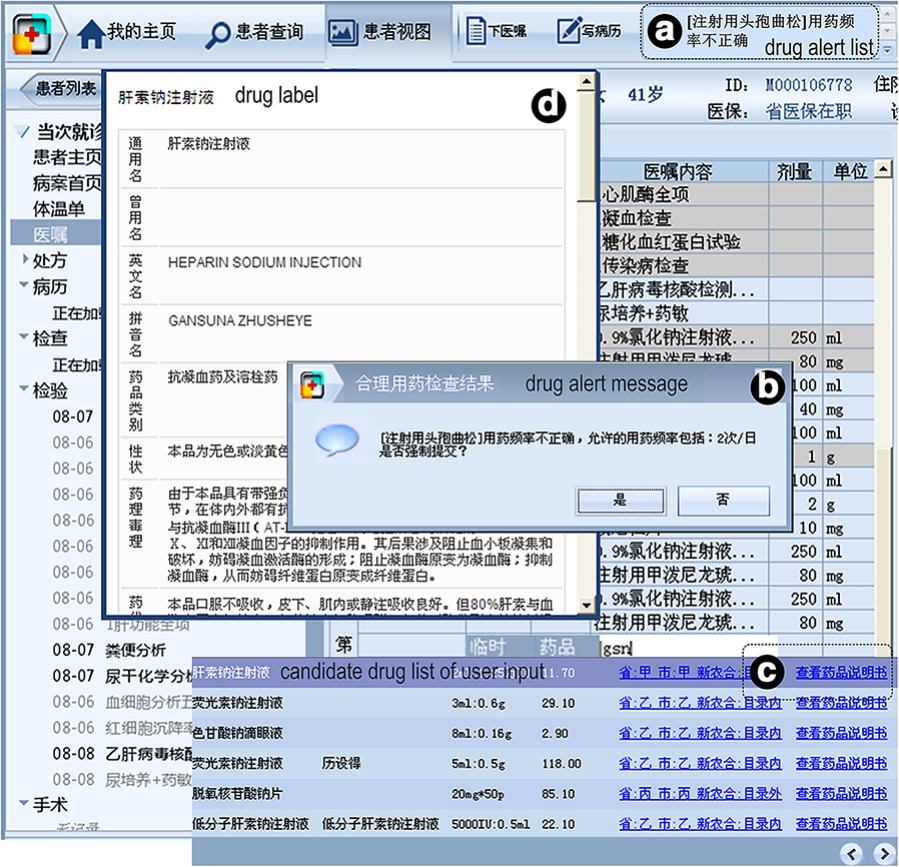
Medication-related clinical decision support in CPOE. **a** Notification area for drug alerts. User can review and process all triggered drug alerts in this area. **b** Drug alert message. **c** Infobutton for drug labels. **d** Retrieved drug label by Infobutton

With the rule set, a medication-related clinical decision support was developed and integrated into CPOE (Fig. 3). Reasoning of the rules is executed by a home-grown rule engine (refer to http://ktp.brahma.top/Display/TestRuleEngine, http://ktp.brahma.top/Pages/Evaluation/RuleEngine/Index.html). The CPOE was also developed by our research team, under the product name “MIAS (Medical Information Automation System)”. The interaction between CPOE and CDS was implemented by web services. Whenever the physician submits orders, CPOE will call the drug checking web service of CDS to trigger the rule engine. CDS-detected alerts are then returned to CPOE, and CPOE displays them to the physician as warnings (Fig. 3b). The physician can either cancel order submission or override the alert. All detected alerts are also sent to the notification area (Fig. 3a) for review. In exceptional cases due to patient status, physicians may state their reasons for overriding the alert. While reviewing the drug alerts, physicians can use infobutton (Fig. 3c) to retrieve related drug labels (Fig. 3d). For pharmacists, we provide a backend web portal for viewing the status (accepted or overridden) and override reason for each alert. The information flow of drug alert status is automatically directed and tracked by the system, which has greatly reduced the necessity of face-to-face communication and telephone calls between physicians and pharmacists.

In this system, only physicians have the right to change the status of an alert (accept or override). Pharmacist only have read-only rights for alert statuses, but they can edit (increase threshold or change rule content) or deactivate corresponding rules if they found many occurrences of an unreasonable alert.

### Evaluation of the rule set in CPOE

The computerized rule set was first implemented in the inpatient CPOE on 2013/12/25 (The outpatient CPOE was provided by another vendor, and had not been integrated with our system). Until now, the system has been used in 49 inpatient departments for more than 2 years. In order to evaluate the actual effect of the rule set, system log data between 2013/12/25 and 2015/07/01 were collected. During this period, totally 68,182 inpatient visits were enrolled into the system and 2,747,140 medication orders were submitted.

For the submitted medication errors, totally 18,666 alerts were detected by the CDS, and 2803 alerts were overridden by physicians. Therefore, the overall override rate is 15.0% (2803/18,666), and accept rate is 85%. Among the 18,666 alerts, Chinese patent medicine (CPM) takes up 38.4% (7168 in 18,666).

According to Tables 2 and 3, several results caught our attention and we further analyzed these results.

**Table 2:** Drug alert analysis

Drug name	Drug name (Chinese)	Alert type	Alerts	Overridden alerts	Override rate (%)
Ambroxol injection	氨溴索注射液	Daily dosage	4938	22	0.4
Salvia TMP injection	丹参川芎嗪注射液	Daily dosage	4039	0	0.0
Injection esomeprazole	注射用埃索美拉唑	Dissolvent dosage	1261	1239	98.3
Thin Chi glycopeptide injection	薄芝糖肽注射液	Daily dosage	1050	2	0.2
Shuxuening injection	舒血宁注射液	Daily dosage	876	0	0.0
Fufangkushen injection	复方苦参注射液	Daily dosage	761	4	0.5
Lidocaine hydrochloride injection	盐酸利多卡因注射液	Skin test	691	287	41.5
Injection cefathiamidine	注射用头孢硫脒	Daily dosage	488	0	0.0
Injection thymopentin	注射用胸腺五肽	Administration route	413	277	67.1
Calcium gluconate injection	葡萄糖酸钙注射液	Dissolvent	307	0	0.0
Iron sucrose injection	蔗糖铁注射液	Dissolvent dosage	298	0	0.0
Injection ambroxol	注射用氨溴索	Administration route	248	0	0.0
Injection aminophylline	氨茶碱注射液	Dissolvent	229	161	70.3
Injection pantoprazole	注射用泮托拉唑	Dissolvent dosage	219	111	50.7
Yinxingdamo injection	银杏达莫注射液	Dissolvent dosage	203	102	50.2
Injection omeprazole	注射用奥美拉唑	Administration route	198	191	96.5
Injection pantoprazole	注射用泮托拉唑	Administration route	133	46	34.6
Injection of fat-soluble vitamins II	注射用脂溶性维生素II	Dissolvent	131	10	7.6
Ceftriaxone for injection	注射用头孢曲松	Frequency	116	56	48.3
Injection cefamandole ester	注射用头孢孟多酯	Prescription restriction	113	0	0.0
Injection pancreatic kallikrein	注射用胰激肽原酶	Administration route	113	0	0.0
Leucovorin injection	亚叶酸钙注射液	Administration route	112	0	0.0
Injection cefoxitin	注射用头孢西丁	Prescription restriction	110	0	0.0
Injection omeprazole	注射用奥美拉唑	Dissolvent dosage	103	61	59.2
Oxytocin injection	缩宫素注射液	Dissolvent	96	0	0.0
Heparin sodium injection	肝素钠注射液	Administration route	91	0	0.0
Sodium for injection cefodizime	注射用头孢地嗪钠	Prescription restriction	87	0	0.0
Alprostadil injection	前列地尔注射液	Administration route	80	28	35.0
Furosemide injection	呋塞米注射液	Dissolvent	70	51	72.9
Injection esomeprazole	注射用埃索美拉唑	Frequency	60	0	0.0
Salvia TMP injection	丹参川芎嗪注射液	Dissolvent dosage	57	0	0.0
Injectable piperacillin sodium and tazobactam sodium	注射用哌拉西林钠他唑巴坦钠	Prescription restriction	53	0	0.0
Cefoperazone sulbactam	注射用头孢哌酮舒巴坦	Prescription restriction	51	0	0.0
Kangai injection	康艾注射液	Dissolvent dosage	47	0	0.0
Leucovorin injection	亚叶酸钙注射液	Frequency	43	0	0.0
Levofloxacin injection	左氧氟沙星注射液	Dissolvent dosage	38	21	55.3
Injection torasemide	注射用托拉塞米	Frequency	38	0	0.0
Large plants Rhodiola injection	大株红景天注射液	Dissolvent dosage	37	0	0.0
Cefoperazone	注射用头孢哌酮	Prescription restriction	36	0	0.0
Xuebijing injection	血必净注射液	Dissolvent dosage	36	26	72.2
Injection of fat-soluble vitamins II	注射用脂溶性维生素II	Daily dosage	33	3	9.1
Ceftazidime for injection	注射用头孢他啶	Prescription restriction	30	0	0.0
Injection imipenem cilastatin sodium	注射用亚胺培南西司他丁钠	Prescription restriction	28	0	0.0
Sodium for injection aescinate	注射用七叶皂苷钠	Daily dosage	24	3	12.5
Torasemide injection	托拉塞米注射液	Frequency	23	0	0.0
Shuxuening injection	舒血宁注射液	Dissolvent	21	17	81.0
Injection of water-soluble vitamins	注射用水溶性维生素	Dosage	21	0	0.0
Amiodarone injection	胺碘酮注射液	Dissolvent	20	15	75.0
Injection ulinastatin	注射用乌司他丁	Frequency	20	0	0.0
Meropenem for injection	注射用美罗培南	Prescription restriction	19	0	0.0
Polyene phosphatidylcholine injection	多烯磷脂酰胆碱注射液	Dissolvent	19	11	57.9
Injection pantoprazole	注射用泮托拉唑	Dissolvent	18	8	44.4
Insulin injection	胰岛素注射液	DDI	17	3	17.6
Fluconazole injection	氟康唑注射液	Prescription restriction	16	0	0.0
Injection esomeprazole	注射用埃索美拉唑	Dosage	15	0	0.0
Sodium for injection aescinate	注射用七叶皂苷钠	Dosage	15	0	0.0
Vancomycin injection	注射用万古霉素	Prescription restriction	14	0	0.0
Vitamin C injection	维生素C注射液	DDI	13	3	23.1
Injection omeprazole	注射用奥美拉唑	Dissolvent	13	12	92.3
Methylprednisolone sodium succinate injection	注射用甲泼尼龙琥珀酸钠	DDI	11	1	9.1
Injection carbazochrome sodium sulfonate	注射用卡络磺钠	Dissolvent	11	7	63.6
Itraconazole oral solution	伊曲康唑口服液	Prescription restriction	10	0	0.0
Fufangkushen injection	复方苦参注射液	Dosage	10	0	0.0
Flurbiprofen injection	氟比洛芬酯注射液	Dosage	10	0	0.0
Injection lentinan	注射用香菇多糖	Dosage	10	0	0.0
Other low occurrence drug alerts (i.e. fired alert count <10)			155	25	16.1
Total			18,666	2803	15.0

**Table 3:** drug alert analysis grouped by rule types

Alert type	Alerts	Overridden alerts	Override rate (%)
Daily dosage	12,212	34	0.3
Dissolvent dosage	2299	1560	67.9
Administration route	1391	542	39.0
Dissolvent	964	312	32.4
Skin test	691	287	41.5
Prescription restriction	595	0	0.0
Frequency	300	56	18.7
Dosage	151	5	3.3
DDI	63	7	11.1
Total	18,666	2803	15.0

1. Among the detected alerts, “daily dosage” rule type has the highest alert occurrence rate (12,212 alerts in total 18,666). We dived into the “daily dosage” alerts, and found four of the top five drugs are CPM, i.e. “Salvia TMP injection (4039 alerts)”, “Thin Chi glycopeptide injection (1050 alerts)”, “Shuxuening injection (876 alerts)” and “Fufangkushen Injection (761 alerts)”, which are responsible for the majority of “daily dosage” alerts. CPM is mostly extracted or manufactured from Chinese traditional herbs. Compared to western synthesized chemical medicine, though herbs take much longer time to take effect, they also have fewer side effects and adverse reactions. In fact, CPM usually plays an auxiliary or supportive role in treatment regimens. For this reason, some physicians relaxed their vigilance and didn’t pay enough attention when using CPM. This also explains why CPM has a noticeable percentage in all the detected alerts (38.4%).
2. The “dissolvent dosage” rule type has the highest override rate (67.9%). The 67.9% override rate is remarkably high compared to other rule types, which means about 2/3 “dissolvent dosage” alerts have been overridden. We consulted with the clinical pharmacists, and found many alerts were related to patients with certain conditions, e.g. renal deficiency or heart failure. For such patients, it is reasonable to use smaller dosage than required by the drug fact sheet. Such false-positive cases have added up to the overridden alerts. To address this issue, we are currently considering using more patient context data to exclude such false-positive alerts.
3. The “skin test” rule type has the second highest override rate (41.5%). Investigation reveals that this high override rate is caused by the discrepancy in physicians’ understanding of the “skin test” rule. In this system, the skin test rule is not designed as a mandatory requirement for the current specific patient, but a general risk reminder for nurses. That means, if there is potential allergic risk (either from medical literature or drug fact sheet) for a certain drug, physicians should set the skin test flag for corresponding medication orders. If not, the skin test rule will give an alert. When it comes to the drug administrating phase, the nurses will investigate this flag as well as patient’s specific conditions (e.g. known allergy history towards certain drugs) to judge whether skin test is needed. However, many physicians treated the “skin test” rule as patient-specific flags, i.e. if a certain drug has potential allergic risk, but the physician already knows the current patient is not allergic to this drug, he/she will not set the flag and override the skin test alert.

Besides the above analysis for certain rule types, there are also high alert occurrence and override rates for several individual drugs, which are caused by different reasons and need case-by-case investigation. Base on these periodical retrospective analyses, pharmacists can continually improve the rule set (e.g. change threshold, revise rule content, deactivate rules) to better suit clinical use.

## Discussion

The primary contribution of this study is a concise drug alerting rule set oriented to Chinese hospitals. As the rule set was built based on the historical data from a large-scale (2600-bed) general hospital with high patient throughput (e.g. 68,182 inpatient visits from 2013/12/25 to 2015/07/01), the rule set should be able to reflect the medication use profile of large populations and may serve as a reference for other Chinese hospitals.

In this study, the computerized rule set can be used as a “preliminary screening tool” against physicians’ medication orders. In DaYi Hospital, pharmacists need to check 4968 medication orders per day on average, and unqualified orders have to be returned to physicians. This is a time-consuming and laborious work. With the drug alerting CDS, many potential mistakes can be ruled out before they reach the final checkpoint of pharmacists. According to the evaluation result, physicians have revised 85% of detected medication orders. In the long run, the system will not only alleviate the workload of pharmacists (many drug use errors can be revised by the physicians without pharmacists’ intervention) but also enhance the workflow efficiency (avoid the “reject-revise-resubmit” process).

This study has several limitations or arguments:

1. The proposed rule set is not suitable for procedural drug rules. For example, the preparation of azithromycin solution is a multi-step procedure. First, azithromycin is dissolved with sterilized water to formulate into 0.1 g/ml. Then, add it to 250–500 ml 0.9% NaCl or 5% glucose solution to get a 1.0–2.0 mg/ml concentration. This procedural logic cannot be easily represented as a single succinct dissolvent rule.
2. The current rule set doesn’t support complex personalized dosing algorithms. In certain contexts, such as children or elder patients with different body weights and body surface areas, or patients with renal insufficiency based on creatinine clearance, more complicated personalized dosing algorithms are needed. To support them, the information model needs further extension to represent such individualized knowledge.
3. DDI rule subtyping. In current system implementation, all DDI rules are treated as one rule type. However, it’s better to design more DDI sub-types in order to achieve more fine-grained alerts. For example, the SFINX project (Andersson et al. 2015) tiers DDIs according to clinical significance (A–D), which enables fine-grained threshold settings for automated warnings.
4. Lack of complete evaluation. In this study, the accept and override rates can be easily calculated from the log data. However, it is not so easy to calculate accuracy and specificity, which requires reviewing every overridden alert in order to identify true positives and false positives. In the future, we will build a “closed-looped” alert tracking workflow, in which the state changes (either by physicians or pharmacists) and change reasons (e.g. why physician override an alert, and why pharmacists reject overriding an alert) of each alert are tracked and logged by the system.
5. Use of clinically identified ADEs. ADEs (adverse drug events) are valuable data for analyzing drug use and medication-related CDS. In China, we have a multi-level ADE reporting mechanism. Level I: Physicians submit detected ADE and related clinical data (patient demographics, symptoms, drug use info, etc.) to the hospital’s pharmacy department. Level 2: Pharmacists submit confirmed ADEs to drug regulatory authorities, i.e. China SFDA (a counterpart of US FDA). Level 3: China SFDA evaluates drug risks based on nation-wide collected ADEs. Although this ADE-reporting mechanism is well designed, it’s a sad reality that it hasn’t lived up to its maximum benefit, largely due to the wide-spread under-reporting problems. Most ADE events were concealed or neglected in daily practices, and the few reported ADEs cannot be used as a solid and complete data source for analyzing physicians’ drug use and evaluating our rule set. To address this issue, we are currently cooperating with clinical pharmacists to detect unreported ADEs from clinical documents (e.g. patient daily progress notes) by natural language processing (NLP) technologies.
6. Coverage of the rule set. One basic assumption of this study is that drug alerts conform to Pareto-alike distribution, where small portion of drug rules accounts for the majority of alerts. As a supporting case, one US study in 2005 (Reichley et al. 2005) used a commercial drug alerting rule set. It contains 48,262 rules for 1537 drugs, but 90% of alerts are focused on 58 drugs. From their daily work experience, the pharmacists in DaYi hospital also hold the same opinion that small set of drugs generate majority of errors. However, to further verify this assumption, a further evaluation is needed to get the coverage rate of the rule set. This requires a full set for all drugs on the Chinese market, and a parallel comparison of the full set and concise set on a large-scale and long-term patient drug use data set. A coverage rate greater than 80% should be ideal. Otherwise, more rules may have to be added to the rule set.
7. Another problem of the rule set is how to keep up with the latest clinical evidence. Occasionally published guidelines or case reports will necessitate adding or revising rules. For example, the China SFDA (State Food and Drug Administration) periodically publish ADE (adverse drug events) reports collected all round the country. A well-maintained rule set should keep up with these public sources. Currently, our research team is developing a semi-automatic program based on NLP, which will help pharmacists extract structured contents from the public ADE reports.

Generally speaking, the overall 85.0% accept rate indicates the rule set has been well received by physicians [compared to the override rates reported in other recent studies, e.g. 53.6% (Nanji et al. 2014), 87.6% (Topaz et al. 2015)] and is effective in reducing pharmacists’ workload. Moreover, the pharmacists are continually analyzing (i.e. analyze those drug alerts with high override rates), improving (e.g. raise alert threshold to reduce false positive alerts) and expanding (i.e. add more drugs and rules) the drug rule set, which will further improve its accuracy and coverage. However, due to the various complex and individualized patient statuses, such a computerized rule set is never meant to substitute the routine work of pharmacists, but can be used an effective supportive tool.

## Conclusions

 In this study, a concise drug alerting rule set for Chinese hospitals was constructed by pharmacists. The case study in a Chinese hospital indicates the medication-related CDS based on the rule set has been well received by physicians. For other hospitals, they may use this rule set as a starter kit for building their own medication-related CDS systems or use it to tier commercial rule bases.

## Appendix

See Tables 4 and 5.

**Table 4:** Drug rule set (Part 1)

Drug	Drug (Chinese name)	Rule content	Rule type
Compound matrine injection^a^	复方苦参注射液^a^	2–3 dose	Dosage
Injection aescinate^a^	注射用七叶皂苷钠^a^	10–20 mg	Dosage
Injection of Ginkgo biloba extract^a^	银杏叶提取物注射液^a^	≤4 dose	Dosage
Salvia ligustrazine injection^a^	丹参川芎嗪注射液^a^	≤2 dose	Dosage
Ambroxol injection	盐酸氨溴索注射液	≤2 dose	Dosage
Phosphate sodium for injection	磷酸钠注射液	≤2 dose	Dosage
Thin chi glycopeptide injection^a^	薄芝糖肽注射液^a^	≤2 dose	Dosage
Citicoline injection	胞二磷胆碱注射液	≤2 dose	Dosage
Tiopronin injection	硫普罗宁注射液	≤2 dose	Dosage
Injection cefathiamidine	注射用头孢硫脒	≤1 dose	Dosage
ShuXueNing injection^a^	舒血宁注射液^a^	≤2 dose	Dosage
Injection esomeprazole	注射用埃索美拉唑	20–40 mg	Dosage
Flurbiprofen injection	氟比洛芬酯注射液	50 mg	Dosage
Injection lentinan	注射用香菇多糖	1 mg	Dosage
Hydrocortisone injection	氢化可的松注射液	50–100 mg	Dosage
Xiaoaiping injection^a^	消癌平注射液^a^	2–4 ml	Dosage
Calcium gluconate injection	葡萄糖酸钙注射液	1–2 g	Dosage
Sodium phosphate injection	注射用磷酸肌酸钠	≤1 dose	Dosage
Javanica oil emulsion injection^a^	鸦胆子油乳注射液^a^	10–30 ml	Dosage
Injection ulinastatin	注射用乌司他丁	100,000U	Dosage
Injection of Ginkgo biloba extract^a^	银杏叶提取物注射液^a^	5 dose	Daily dosage
Compound matrine injection^a^	复方苦参注射液^a^	3 dose	Daily dosage
Salvia ligustrazine injection^a^	丹参川芎嗪注射液^a^	2 dose	Daily dosage
Ambroxol injection	盐酸氨溴索注射液	2 dose	Daily dosage
Phosphate sodium for injection	磷酸钠注射	2 dose	Daily dosage
Thin chi glycopeptide injection^a^	薄芝糖肽注射液^a^	2 dose	Daily dosage
Citicoline injection	胞(二)磷胆碱注射液	2 dose	Daily dosage
Tiopronin injection	硫普罗宁注射液	2 dose	Daily dosage
Injection aescinate^a^	注射用七叶皂苷钠^a^	20 mg	Daily dosage
Injection cefathiamidine	头孢硫脒注射液	1 dose	Daily dosage
Injection of fat-soluble vitamins I	注射脂溶性维生素I	1 dose	Daily dosage
Injection of fat-soluble vitamins II	注射脂溶性维生素II	1 dose	Daily dosage
Injection of water-soluble vitamins	注射用水溶性维生素	1 dose	Daily dosage
L-carnitine injection	左旋肉碱注射液	Iv push	Administration route
Omeprazole injection (Losec)	奥美拉唑注射液 (罗塞克)	Iv push	Administration route
Omeprazole injection (AoXiKang, Luoren)	奥美拉唑注射液 (奥西康,罗润)	Iv drip	Administration route
Injection thymopentin	注射用胸腺五肽	Intramuscular injection, subcutaneous injection	Administration route
Injection cobamamide	注射用腺苷钴胺	Intramuscular injection	Administration route
Ambroxol injection	盐酸氨溴索注射液	Iv	Administration route
Furosemide injection	呋塞米注射液	Iv	Administration route
Pantoprazole injection	注射用泮托拉唑	Iv drip	Administration route
Pancreatic kininogenase for injection	注射用胰激肽原酶	Intramuscular injection	administration route
Leucovorin injection	亚叶酸钙注射液	Iv drip	Administration route
L-carnitine injection	左卡尼汀注射液	Iv	Administration route
Heparin sodium injection	肝素钠注射液	Subcutaneous injection, iv	Administration route
Alprostadil injection	前列地尔注射液	Iv	Administration route
Ceftriaxone for injection	头孢曲松钠注射液	1/day	Frequency
Injection esomeprazole	注射用埃索美拉唑	1/day, 1/12 h	Frequency
Leucovorin injection	亚叶酸钙注射液	1/day, 1/6 h	Frequency
Injection torasemide	注射用托拉塞米	1/day	Frequency
Torasemide injection	托拉塞米注射液	1/day	Frequency
Injection ulinastatin	注射用乌司他丁	1–3/day	Frequency
Lidocaine hydrochloride injection	盐酸利多卡因注射液	Skin test required	Skin Test
Furosemide injection	呋塞米注射液	NaCl, sterile water	Dissolvent
Sodium heparin injection	肝素钠注射液	NaCl, sterile water	Dissolvent
Brain carnosine injection	脑肌肽注射液	NaCl, 5% glucose	Dissolvent
Tanreqing injection^a^	痰热清注射液^a^	NaCl, 5% glucose	Dissolvent
Pantoprazole injection	注射用泮托拉唑	NaCl	Dissolvent
Injection carbazochrome sodium sulfonate	注射用卡络磺钠	NaCl	Dissolvent
Edaravone injection	依达拉奉注射液	NaCl	Dissolvent
Lipoic acid injection	硫辛酸注射液	NaCl	Dissolvent
Xuebijing injection^a^	血必净注射液^a^	NaCl	Dissolvent
Iron sucrose injection	蔗糖铁注射液	NaCl	Dissolvent
Injection of fat-soluble vitamins I	注射脂溶性维生素I	Glucose, sterile water	Dissolvent
Injection of fat-soluble vitamins II	注射脂溶性维生素II	Glucose, sterile water	Dissolvent
Paclitaxel liposome for injection	紫杉醇脂质体注射液	Glucose, sterile water	Dissolvent
Injection of liposomal amphotericin B	注射用两性霉素B脂质体	Glucose, sterile water	Dissolvent
Polyene phosphatidylcholine injections	多烯磷脂酰胆碱注射液	Glucose, sterile water	Dissolvent
Injection breviscapine^a^	注射用灯盏花素^a^	5% glucose, 10% glucose, 0.9% NaCl	Dissolvent
Aminophylline injection	氨茶碱注射液	Glucose	Dissolvent
Injection of Ginkgo biloba extract^a^	银杏叶提取物注射液^a^	Glucose	Dissolvent
Amiodarone injection	胺碘酮注射液	5% glucose	Dissolvent
Injection pHGF	注射用促肝细胞生长素	10% glucose	Dissolvent
Ginkgo leaf extract and dipyridamole injection^a^	银杏达莫注射液^a^	0.9% NaCl, 5% glucose, 10% glucose	Dissolvent
Omeprazole injection	奥美拉唑注射液	0.9% NaCl	Dissolvent
Calcium gluconate injection	葡萄糖酸钙注射液	10% glucose	Dissolvent
Oxytocin injection	缩宫素注射液	NaCI	Dissolvent
TanReQing injection^a^	痰热清注射液	5% glucose	Dissolvent
ShuXueNing injection^a^	舒血宁注射液^a^	5% glucose	Dissolvent
Ginkgo leaf extract and dipyridamole Injection^a^	银杏达莫注射液^a^	500 ml	Dissolvent dosage
Levofloxacin	左氧氟沙星	250 ml	Dissolvent dosage
Pantoprazole injection	注射用泮托拉唑	100 ml	Dissolvent dosage
Xuebijing injection^a^	血必净注射液^a^	100 ml	Dissolvent dosage
Iron sucrose injection	蔗糖铁注射液	≤100 ml	Dissolvent dosage
Injection esomeprazole	注射用埃索美拉唑	100 ml	Dissolvent dosage
Omeprazole injection	奥美拉唑注射液	100 ml	Dissolvent dosage
Salvia ligustrazine injection^a^	丹参川芎嗪注射液^a^	250–500 ml	Dissolvent dosage
Large plants Rhodiola injection^a^	大株红景天注射液^a^	250 ml	Dissolvent dosage
Triazolam tablets	三唑仑片	FDA pregnancy category X—use on pregnant women is forbidden	Pregnancy risk
Ribavirin	利巴韦林		Pregnancy risk
Estradiolvalerate	戊酸雌二醇片		Pregnancy risk
Fluorouracil Injection	氟尿嘧啶注射液		Pregnancy risk
Misoprostol tablets	米索前列醇片		Pregnancy risk
Simvastatin	辛伐他汀		Pregnancy risk
Avi A capsules	阿维A胶囊		Pregnancy risk
Estazolam tablets	艾司唑仑片		Pregnancy risk
Bicalutamide tablets	比卡鲁胺片		Pregnancy risk
Goserelin acetate sustained-release implants	醋酸戈舍瑞林缓释植入剂		pregnancy risk
Finasteride tablets	非那雄胺片		Pregnancy risk
Fluvastatin	氟伐他汀		Pregnancy risk
Fluorouracil implants	氟尿嘧啶植入剂		Pregnancy risk
Mifepristone misoprostol tablets	米非司酮米索前列醇片		Pregnancy risk
Levonorgestrel	左炔诺孕酮		Pregnancy risk
Estradiol	雌二醇		Pregnancy risk
Injection cefamandole ester	注射用头孢孟多酯	Restricted antibiotics. Only chief physicians and above have the prescription right. Resident physicians cannot directly prescribe these drugs	Prescription restriction
Injection cefoxitin	注射用头孢西丁		Prescription restriction
Sodium for injection cefodizime	注射用头孢地嗪钠		Prescription restriction
Injectable piperacillin sodium and tazobactam sodium	注射用哌拉西林钠他唑巴坦钠		Prescription restriction
Cefoperazone sulbactam	注射用头孢哌酮舒巴坦		Prescription restriction
Cefoperazone	注射用头孢哌酮		Prescription restriction
Ceftazidime for injection	注射用头孢他啶		Prescription restriction
Injection imipenem cilastatin sodium	注射用亚胺培南西司他丁钠		Prescription restriction
Meropenem for injection	注射用美罗培南		Prescription restriction
Fluconazole injection	氟康唑注射液		Prescription restriction
Vancomycin injection	注射用万古霉素		Prescription restriction
Itraconazole oral solution	伊曲康唑口服液		Prescription restriction
Minocycline hydrochloride capsules	盐酸米诺环素胶囊		Prescription restriction
Moxifloxacin injection	莫西沙星注射液		Prescription restriction
Injectable piperacillin sulbactam	注射用哌拉西林舒巴坦		Prescription restriction
Injection voriconazole	注射用伏立康唑		Prescription restriction
Azithromycin for injection	注射用阿奇霉素		Prescription restriction
Injection caspofungin	注射用卡泊芬净		Prescription restriction
Injection teicoplanin	注射用替考拉宁		Prescription restriction
Linezolid injection	利奈唑胺注射液		Prescription Restriction
Moxifloxacin tablets	莫西沙星片		Prescription restriction
Injection of amphotericin B liposome	注射用两性霉素B脂质体		Prescription restriction

^a^Chinese patent medicine

**Table 5:** Drug rule set (Part 2: DDI pairs)

Drug 1		Drug 2		Description
Vitamin C injection	维生素C注射液	Vitamin K1 injection	维生素K1注射液	Mixture prone to turbidity^a^
Four-vitamin injection	复方维生素注射液(4)	Injection of fat-soluble vitamins I	注射脂溶性维生素我	Same ingredient. Duplicate therapy^a^
		Injection of fat-soluble vitamins II	注射脂溶性维生素II	
Methylprednisolone sodium succinate for injection	甲泼尼龙琥珀酸钠注射液	Insulin injection	胰岛素注射	Methylprednisolone sodium succinate for injection increases requirements for insulin or oral hypoglycemic agents in diabetics^a^
Methylprednisolone sodium succinate for injection	甲泼尼龙琥珀酸钠注射液	Recombinant human insulin injection	重组人胰岛素注射	
Methylprednisolone sodium succinate for injection	甲泼尼龙琥珀酸钠注射液	Protamine recombinant human insulin injection	鱼精蛋白重组人胰岛素注射液	
Salvia ligustrazine injection^b^	丹参川芎嗪注射液^b^	Vitamin K1 Injection	维生素K1注射液	Antagnistic effect^a^
Ginkgo biloba extract injection	银杏叶提取物注射液^b^	injection calf blood protein extract	注射用小牛血去蛋白提取物	Cause serious adverse effects, such as gastrointestinal discomfort, headache, decreased blood pressure, allergic reactions
Selegiline	司来吉兰	Pseudoephedrine	伪麻黄碱	MAO inhibitors—Amphetamine and derivatives
		Diethylpropion	二乙胺	
		Fluoxetine	氟西汀	MAO inhibitors— selective serotonin reuptake inhibitors (SSRIs)
		Paroxetine	帕罗西汀	
		Citalopram	西酞普兰	
		Escitalopram	艾司西酞普兰	
		Sertraline	舍曲林	
		Fluvoxamine	氟伏沙明	
		Duloxetine	度洛西汀	
		Venlafaxine	文拉法辛	
		Meperidine	哌替啶	MAO inhibitors—narcotic analgesics
		Fentanyl	芬太尼	
		Tramadol	曲马多	
		Amitriptyline	阿米替林	Selegiline—tricyclic antidepressants (TCAs)
Irinotecan	伊立替康	Clarithromycin	克拉霉素	Irinotecan—strong CYP3A4 inhibitors
		Erythromycin	红霉素	
		Amiodarone	胺碘酮	
		Verapamil	维拉帕米	
		Diltiazem	地尔硫卓	
		Ketoconazole	酮康唑	
		Itraconazole	伊曲康唑	
		Fluconazole	氟康唑	
		Voriconazole	伏立康唑	
		Cimetidine	西咪替丁	
Simvastatin	辛伐他汀	Clarithromycin	克拉霉素	HMG Co-A reductase inhibitors—CYP3A4 inhibitors
		Erythromycin	红霉素	
		Amiodarone	胺碘酮	
		Verapamil	维拉帕米	
		Diltiazem	地尔硫卓	
		Ketoconazole	酮康唑	
		Itraconazole	伊曲康唑	
		Fluconazole	氟康唑	
		Voriconazole	伏立康唑	
		Roxithromycin	罗红霉素	Severe DDI reported from literature, including rhabdomyolysis and liver damage
Ergotamine	麦角胺	Clarithromycin	克拉霉素	Ergot alkaloids and derivatives—CYP3A4 inhibitors
		Erythromycin	红霉素	
		Ketoconazole	酮康唑	
		Itraconazole	伊曲康唑	
		Voreconazole	伏立康唑	
Tizanidine	替扎尼定	Ciprofloxacin	环丙沙星	Tizanidine—CYP1A2 inhibitors
		Fluvoxamine	氟伏沙明	
		Mexiletine	美西律	
		Propafenone	普罗帕酮	
		Amiodarone	胺碘酮	
Zolmitriptan	佐米曲普坦	Moclobamide	吗氯贝胺	Triptans—MAO inhibitors
		Methylene blue	亚甲蓝	
Chloroquine	氯喹	QT prolonging agents. Any of two drugs have synergistic effect		
Moxifloxacin	莫西沙星			
Sotalol	索他洛尔			
Clarithromycin	克拉霉素			
Citalopram	西酞普兰			
Amiodarone	胺碘酮			
Erythromycin	红霉素			
Haloperidol	氟哌啶醇			
Droperidol	氟哌利多			
Domperidone	多潘立酮			
Procainamide	普鲁卡因胺			
Sevoflurane	七氟醚			
Chlorpromazine	氯丙嗪			
Arsenic trioxide	白砒			
Azithromycin	阿奇霉素			

^a^Means these rules are added by the local pharmacists; others are from the work published by other researchers (Phansalkar et al. 2012)

^b^Chinese patent medicine
